# Associations of eosinophil-to-monocyte ratio and C-reactive protein-to-high-density lipoprotein cholesterol ratio with early neurological deterioration after thrombolysis in acute ischemic stroke

**DOI:** 10.3389/fneur.2025.1687536

**Published:** 2026-01-20

**Authors:** Guohua He, Guilan Xu, Yanqiao Xiao, Zhen Wang, Yachun Yu

**Affiliations:** Department of Neurology, The Affiliated Changsha Central Hospital, Hengyang Medical School, University of South China, Changsha, China

**Keywords:** acute ischemic stroke, intravenous thrombolysis, eosinophil-to-monocyte ratio, CRP/HDL-C ratio, early neurological deterioration

## Abstract

**Background:**

Inflammation mechanisms play critical roles in acute ischemic stroke (AIS). However, the correlations of the eosinophil-to-monocyte ratio (EMR) and blood C-reactive protein to high-density lipoprotein cholesterol (CRP/HDL-C) ratio with post-thrombolysis early neurological deterioration (END) in patients with AIS remain uncertain.

**Methods:**

Patients with AIS who received intravenous thrombolysis therapy from January 2020 to February 2025 were retrospectively recruited for this study. CRP level, blood lipid concentrations, and complete blood count measurements were recorded within 24 h of admission. Post-thrombolysis END was defined as an increase in the U.S National Institutes of Health Stroke Scale (NIHSS) score of ≥ 4 points compared to the initial NIHSS score taken within 24 h of initiating intravenous thrombolysis. Multivariate logistic regression modeling was performed to evaluate the correlations of EMR and the CRP/HDL-C ratio to post-thrombolysis END. Receiver operating characteristic (ROC) curves were used to analyze the predictive value of both EMR and the CRP/HDL-C ratio in patients with post-thrombolysis END.

**Results:**

Among 473 recruited patients, 103 (21.78%) were diagnosed with post-thrombolysis END. Patients with END had significantly higher systolic and diastolic blood pressures, white blood cell and monocyte counts, CRP levels, CRP/HDL-C ratios, and NIHSS scores on admission, while their eosinophil counts and EMRs were significantly lower. The multivariate logistic regression analysis indicated that EMR (odds ratio [OR], 0.03 [95% confidence interval (CI) 0.01–0.14]; *p* < 0.001) and CRP/HDL-C (odds ratio, 1.04[95%CI 1.01–1.08]; *p* = 0.025) were independently associated with END after adjusting for potential confounders. The areas under the receiver operating characteristic curve (AUC) for EMR and the CRP/HDL-C ratio were 0.757 (95% CI, 0.709–0.805) and 0.61 (95% CI, 0.545–0.675), respectively.

**Conclusion:**

A lower EMR level and a higher CRP/HDL-C ratio in patients with AIS are independently associated with post-thrombolysis END. EMR and the CRP/HDL-C ratio may be potential biomarkers for post-thrombolysis END.

## 1 Introduction

Acute ischemic stroke (AIS) is one of the leading causes of disability and mortality worldwide (1). Intravenous thrombolysis with recombinant tissue plasminogen activator (r-tPA) can improve functional outcomes by recanalizing blocked vessels, restoring ischemic penumbra perfusion, and narrowing infarct lesions in patients within 4.5 h of AIS onset (2). However, a large proportion of patients do not benefit from thrombolytic therapy, and some even experience neurological deterioration within 24 h after intravenous thrombolysis, known as early neurological deterioration (END) (3). Among affected patients, the deterioration of early neurological function leads to irreversible damage to the ischemic penumbra, which seriously affects their prognosis (4). Therefore, it is of great significance to find convenient and effective laboratory indicators to better evaluate post-thrombolysis END in patients with AIS.

In recent years, a growing number of studies have shown that the inflammatory response plays an important role in the occurrence and development of AIS (5, 6). Peripheral leukocytes, such as neutrophils, monocytes, and lymphocytes, are indispensable inflammatory cells that play a crucial role in the development and progression of atherosclerosis (7). Ratio indices, such as the neutrophil-to-lymphocyte ratio (NLR), have been reported as a potential biomarker of the inflammatory process and may serve as a prominent predictor in patients with AIS (8, 9). Moreover, studies have found that NLR is closely related to epileptic seizures (10), glioma grading, and prognosis (11). In neurodegenerative diseases such as Parkinson’s disease, it can serve as a biomarker for risk prediction and disease (12). Furthermore, Natalia et al. found that platelet-to-lymphocyte ratio (PLR) has an important value in the assessment of neuroinflammation in progressive supranuclear palsy (13). A recent study has found that AIS triggers a dramatic decrease in the numbers of circulating eosinophils and an increase in circulating monocytes (14). As a novel biomarker, EMR has been reported to be associated with the prognosis of myocardial infarction and pulmonary embolism (15, 16). Recent studies have found that EMR can serve as a biomarker for predicting asthma exacerbations (17). Furthermore, research suggests that a lower EMR is closely related to a poor 3-month outcome of AIS (18). Nevertheless, the association between EMR and post-thrombolysis END in patients with AIS remains unclear.

C-reactive protein (CRP) is a plasma protein that reflects the inflammatory response during the acute phase (19) and has been implicated in various cerebrovascular and cardiovascular diseases. Research suggests that higher CRP levels are associated with a poor prognosis at 3 months after AIS (19). It is well known that blood lipids such as low-density lipoprotein cholesterol (LDL-C) and total cholesterol (TG) are closely related to cerebral atherosclerosis. Low levels of high-density lipoprotein cholesterol (HDL-C) are one of the main factors of dyslipidemia. HDL-C is considered to have anti-atherosclerotic and anti-inflammatory properties due to its promotion of reverse cholesterol transport (20), which can reduce the incidence of cardiovascular and cerebrovascular diseases (21). A large prospective analysis from China suggested that a 16% reduction in the risk of AIS exists for every 1 mmol/L increase in HDL-C (22). In recent years, the CRP/HDL-C ratio has been widely used in the risk assessment and prognosis prediction of cardiovascular diseases due to its ability to comprehensively reflect the collective inflammatory state and lipid metabolism. In addition, a study revealed that the high-sensitivity CRP/HDL-C ratio is closely related to the poor prognosis of patients with AIS (23). However, the impact of the CRP/HDL-C ratio on the post-thrombolysis END in AIS patients has not yet been clarified.

This study aimed to investigate the associations of EMR and the CRP/HDL-C ratio with post-thrombolysis END, and to explore the predictive value of both EMR and CRP/HDL-C ratio in the post-thrombolysis END among AIS patients.

## 2 Methods

### 2.1 Study design and population

**Figure 1:**
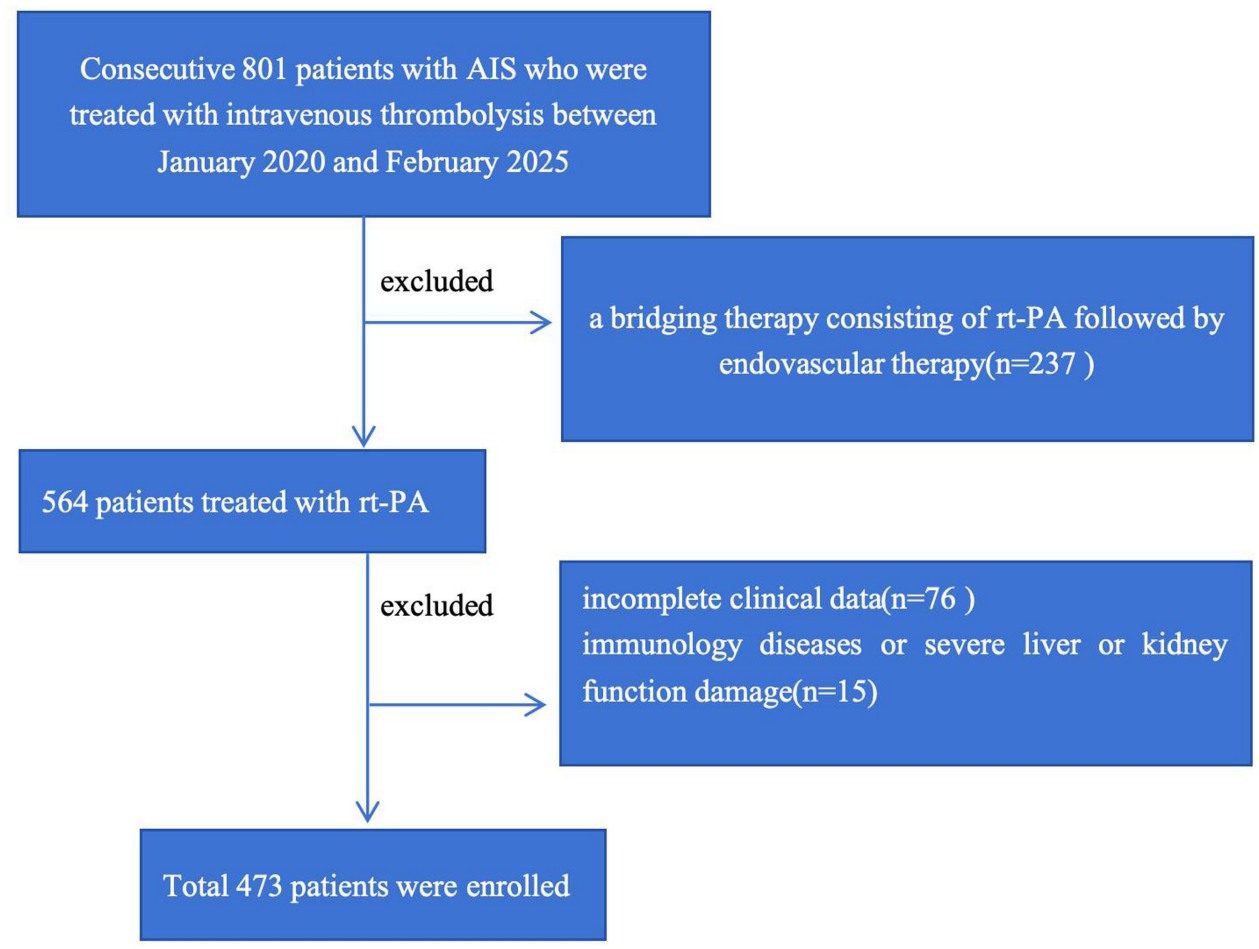
Flow diagram of study population selection. IS, acute ischemic stroke.

As shown in Figure 1, we retrospectively included 473 consecutive patients with AIS who underwent intravenous thrombolysis within 4.5 h of AIS onset at Changsha Central Hospital from January 2020 to February 2025. The inclusion criteria were as follows: (1) AIS was diagnosed according to the 2018 Chinese guidelines for diagnosis and treatment of AIS. (2) Admission occurred within 4.5 h after onset, and patients received intravenous thrombolysis with rt-PA. (3) Patients were aged ≥18 years. Separately, patients were excluded for the following reasons: (1) bridging therapy; (2) severe inflammatory diseases, infectious diseases, or severe hepatic or renal dysfunction; and (3) incomplete clinical data. The study was approved by the Human Research Ethics Committee of Changsha Central Hospital.

### 2.2 Data acquisition

Two authors extracted data related to demographic information (age and sex), vascular risk factors (current smoking, current drinking, hypertension, diabetes mellitus, coronary heart disease, atrial fibrillation, and previous stroke), and other clinical features (blood pressure on admission, NIHSS score on admission, and stroke subtype), and laboratory findings. In addition, none of the enrolled patients had taken medications known to affect white blood cell counts prior to admission. For study purposes, symptomatic intracranial hemorrhage refers to any type of hemorrhagic infarction occurring after 24 h of intravenous thrombolysis with an increase in the NIHSS score of ≥ 4 points (24). In addition, AIS etiology was classified according to the TOAST classification. Considering the relatively small numbers of participants with AIS of either another determined or an unknown etiology, we combined these two groups into one (other causes/unknown).

### 2.3 Variables

Whole-blood samples, CRP levels, and serum lipid concentrations were collected after an 8-h fast on the day following admission. For routine blood tests, 2 mL of EDTA-anticoagulated whole blood was analyzed using an automated hematology analyzer (BZ6800, China). For biochemical assays, 5 mL of coagulant-containing blood was analyzed using an automatic analyzer (HITACHI 7600, Japan). Hematological parameters were measured immediately after blood collection. EMR was calculated using the eosinophil count divided by the monocyte count. The CRP/HDL-C ratio was calculated by dividing the CRP level by the HDL-C level.

### 2.4 Outcome variable evaluation

Post-thrombolysis END was defined as an increase in the National Institutes of Health Stroke Scale (NIHSS) score by ≥ 4 points compared to the initial NIHSS score recorded within 24 h after initiating intravenous thrombolysis, while non-END was defined as a decrease or no change in the NIHSS score. The NIHSS score was evaluated by two professional physicians.

### 2.5 Statistical analysis

Statistical analyses were performed with SPSS 26.0 and GraphPad Prism 9.5.1 software. Continuous variables were expressed as the mean with standard deviation (mean ± SD) or medians (interquartile ranges, IQR), whereas categorical variables were presented as frequencies and percentages. Independent sample *t*-test, Mann–Whitney U-test, one-way analysis of variance (ANOVA), or Kruskal–Wallis test were used appropriately for the comparison of continuous data. In addition, categorical variables were analyzed using the chi-squared test or Fisher’s exact probability method. We used the violin plots to show the distribution of EMR and CRP/HDL-C between END and non-END patients, and data were expressed as mean ± standard error of the mean (SEM). Multivariate logistic regression analysis was performed for statistically significant variables to verify the independent effects of EMR and CRP/HDL-C on END. The ROC curve was used to evaluate the predictive ability of EMR and CRP/HDL-C for post-thrombolysis END. A two-tailed value of *p* < 0.05 was considered significant.

## 3 Results

### 3.1 Baseline characteristics of the study participants

A total of 473 consecutive patients with AIS who received intravenous thrombolytic therapy were included in this study (the participant selection process is illustrated in Figure 1). According to EMR levels, patients were divided into tertiles as follows: T1 (*n* = 156): EMR < 0.24, T2 (*n* = 161):0.24 ≤ EMR ≤ 0.42, and T3 (*n* = 156): EMR > 0.42. The main baseline characteristics of study participants in the aforementioned EMR tertiles are shown in Table 1. Among these three groups, participants with lower EMR values were more significantly female, with a history of hypertension and smoking, and higher TG, albumin, and creatinine levels and NIHSS scores after 24 h of thrombolysis. Moreover, these patients were more likely to have END. Separately, patients were also divided into tertiles according to their CRP/HDL-C ratio as follows: S1 (*n* = 156): CRP/HDL-C ratio <3.17, S2 (*n* = 161):3.17 ≤ CRP/HDL-C ratio ≤4.77, and S3 (*n* = 156): CRP/HDL-C ratio >4.77. The main baseline characteristics of study participants are shown in Table 2. Among them, there were statistical differences in terms of sex, diabetes, CHD, HDL-C, CRP, TG, creatinine, CREA, and NIHSS score on admission and after 24 h of thrombolysis. In addition, patients with higher CRP/HDL-C ratios were more likely to experience END.

**Table 1:** Baseline characteristics of the participants based on EMR tertiles.

Variables	Total (*n* = 473)	EMR < 0.24, (*n* = 156)	0.24 ≤ EMR ≤ 0.42, (*n* = 161)	EMR > 0.42, (*n* = 156)	*p* value
Demographic data					
Age (years)	68 (58–75)	69 (58–77)	66 (58–74)	68 (59–75)	0.166
Sex (male, *n*%)	297 (62.79)	84 (53.85)	103 (63.98)	110 (70.51)	0.009
Vascular risk factors (*n*%)					
Current smoking	187 (39.53)	48 (30.77)	60 (37.27)	79 (50.64)	0.001
Current drinking	87 (18.39)	26 (16.67)	23 (14.29)	38 (24.36)	0.055
Hypertension	305 (64.48)	88 (56.41)	111 (68.94)	106 (67.95)	0.036
Diabetes	99 (20.93)	30 (19.23)	32 (19.88)	37 (23.72)	0.573
Coronary heart disease	58 (12.26)	17 (10.90)	21 (13.04)	20 (12.82)	0.816
Previous stroke	71 (15.01)	22 (14.10)	20 (12.42)	29 (18.59)	0.285
Atrial fibrillation	40 (8.46)	16 (10.26)	13 (8.07)	11 (7.05)	0.582
Stroke subtype, (*n*%)					0.268
Large artery atherosclerosis	125 (26.43)	45 (28.85)	36 (22.36)	44 (28.21)	
Cardioembolism	45 (9.51)	21 (13.46)	12 (7.45)	12 (7.69)	
Small artery occlusion	270 (57.08)	79 (50.64)	102 (63.35)	89 (57.05)	
Other or undetermined	33 (6.98)	11 (7.05)	11 (6.83)	11 (7.05)	
Laboratory data					
WBC (10^9^/L)	7.09 (5.88–8.62)	7.11 (5.76–9.39)	7.06 (5.95–8.33)	7.16 (5.85–8.49)	0.862
Eosinophils (10^9^/L)	0.12 (0.07–0.20)	0.05 (0.03–0.08)	0.13 (0.10–0.16)	0.23 (0.17–0.31)	<0.001
Monocytes (10^9^/L)	0.39 (0.30–0.50)	0.40 (0.30–0.52)	0.41 (0.31–0.49)	0.36 (0.28–0.45)	0.008
HGB (g/L)	139.18 ± 17.89	137.29 ± 19.32	140.32 ± 17.26	139.90 ± 16.97	0.266
TC (mmol/L)	4.41 (3.64–5.11)	4.31 (3.51–5.22)	4.60 (3.75–5.10)	4.38 (3.72–5.04)	0.337
HDL-C (mmol/L)	1.02 (0.88–1.21)	1.04 (0.88–1.20)	1.00 (0.88–1.21)	1.02 (0.90–1.20)	0.788
LDL-C (mmol/L)	2.78 (2.21–3.37)	2.79 (1.97–3.54)	2.85 (2.34–3.39)	2.71 (2.22–3.25)	0.503
TG (mmol/L)	1.39 (0.97–2.09)	1.31 (0.88–1.94)	1.56 (1.04–2.41)	1.33 (0.96–1.91)	0.009
ALB (g/L)	42.00 (39.00–44.00)	42.00 (39.38–44.00)	42.50 (40.00–44.00)	41.00 (39.00–43.00)	0.018
ALT (U/L)	19.00 (14.00–26.00)	18.00 (13.75–26.00)	19.00 (14.00–27.00)	19.00 (14.00–26.00)	0.734
AST (U/L)	22.00 (18.00–27.00)	21.00 (18.00–27.00)	22.50 (18.00–28.00)	22.00 (18.00–27.00)	0.612
Cr (μmol/L)	73.00 (62.00–88.00)	71.50 (61.00–87.25)	73.00 (60.00–85.00)	76.00 (63.00–93.00)	0.043
UA (μmol/L)	333.50 (278.00–400.25)	327.50 (271.50–390.50)	329.00 (280.00–403.00)	345.00 (283.00–394.50)	0.219
Urea (mmol/L)	5.40 (4.56–6.52)	5.37 (4.49–6.56)	5.19 (4.62–6.41)	5.54 (4.55–6.70)	0.553
NIHSS, score on admission	5.00 (3.00–9.00)	6.00 (3.00–10.00)	5.00 (3.00–9.00)	5.00 (3.00–9.00)	0.502
NIHSS score 24 h after starting thrombolysis	5.00 (2.00–9.00)	6.00 (3.00–12.00)	4.00 (2.00–8.00)	4.00 (2.00–7.00)	<0.001
END, (*n*%)	103 (21.78)	64 (41.03)	31 (19.25)	8 (5.13)	<0.001

EMR, eosinophil-to-monocyte ratio; WBC, white blood cell count; HGB, hemoglobin; TC, total cholesterol; TG, triglycerides; HDL-C, high-density lipoprotein cholesterol; LDL-C, low-density lipoprotein cholesterol; ALB, albumin; ALT, alanine aminotransferase; AST, aspartate aminotransferase; Cr, creatinine; UA, uric acid; NIHSS, National Institutes of Health Stroke Scale; END, early neurological deterioration.

**Table 2:** Baseline characteristics of the participants based on CRP/HDL-C ratio tertiles.

Variables	Total (*n* = 473)	CRP/HDL-C < 3.17, (*n* = 156)	3.17 ≤ RP/HDL-C ≤ 4.77, (*n* = 161)	CRP/HDL-C > 4.77, (*n* = 156)	*p* value
Demographic data					
Age (years)	68.00 (58.00–75.00)	68.00 (59.00–75.00)	66.00 (57.00–73.00)	68.00 (59.00–75.25)	0.113
Sex (male, *n*%)	297 (62.79)	82 (52.56)	108 (67.08)	107 (68.59)	0.005
Vascular risk factors (*n*%)					
Current smoking	187 (39.53)	56 (35.90)	71 (44.10)	60 (38.46)	0.310
Current drinking	87 (18.39)	32 (20.51)	28 (17.39)	27 (17.31)	0.706
Hypertension	305 (64.48)	96 (61.54)	108 (67.08)	101 (64.74)	0.586
Diabetes	99 (20.93)	25 (16.03)	30 (18.63)	44 (28.21)	0.021
Coronary heart disease	58 (12.26)	22 (14.10)	11 (6.83)	25 (16.03)	0.031
Previous stroke	71 (15.01)	17 (10.90)	26 (16.15)	28 (17.95)	0.193
Atrial fibrillation	40 (8.46)	11 (7.05)	11 (6.83)	18 (11.54)	0.239
Stroke subtypes, *n*(%)					0.272
Large artery atherosclerosis	125 (26.43)	38 (24.36)	40 (24.84)	47 (30.13)	
Cardioembolism	45 (9.51)	15 (9.62)	10 (6.21)	20 (12.82)	
Small artery occlusion	270 (57.08)	93 (59.62)	100 (62.11)	77 (49.36)	
Other or undetermined	33 (6.98)	10 (6.41)	11 (6.83)	12 (7.69)	
Laboratory data					
WBC (10^9^/L)	7.09 (5.88–8.62)	6.80 (5.80–8.37)	7.07 (5.67–8.42)	7.22 (6.19–9.36)	0.029
HGB (g/L)	139.18 ± 17.89	138.56 ± 15.85	141.37 ± 18.29	137.56 ± 19.23	0.145
TC (mmol/L)	4.41 (3.64–5.11)	4.50 (3.75–5.27)	4.39 (3.58–4.98)	4.26 (3.60–5.23)	0.188
HDL-C (mmol/L)	1.02 (0.88–1.21)	1.21 (1.09–1.35)	0.92 (0.85–1.01)	0.95 (0.79–1.08)	<0.001
LDL-C (mmol/L)	2.78 (2.21–3.37)	2.83 (2.21–3.41)	2.74 (2.22–3.35)	2.77 (2.21–3.44)	0.820
TG (mmol/L)	1.39 (0.97–2.09)	1.17 (0.86–1.64)	1.64 (1.07–2.42)	1.47 (0.99–2.05)	<0.001
CRP	3.41 (3.13–6.09)	3.13 (2.87–3.22)	3.31 (3.19–3.64)	9.21 (5.88–14.10)	<0.001
ALT (U/L)	19.00 (14.00–26.00)	18.50 (14.00–25.00)	19.50 (14.00–26.25)	19.00 (14.00–27.00)	0.730
AST (U/L)	22.00 (18.00–27.00)	22.00 (19.75–26.00)	21.00 (17.00–27.00)	22.00 (18.00–28.00)	0.382
Cr (μmol/L)	73.00 (62.00–88.00)	70.00 (60.00–79.00)	74.00 (63.75–88.00)	78.00 (62.00–98.00)	0.002
UA (μmol/L)	333.50 (278.00–400.25)	313.50 (263.75–381.25)	353.00 (282.75–404.50)	340.00 (288.00–400.25)	0.890
Urea (mmol/L)	5.40 (4.56–6.52)	5.32 (4.50–6.53)	5.44 (4.59–6.49)	5.46 (4.61–6.52)	0.006
SBP (mmHg)	147.00 (134.00–164.00)	145.00 (134.00–165.00)	148.00 (134.00–163.00)	147.00 (135.75–166.00)	0.637
DBP (mmHg)	84.00 (75.00–94.00)	84.00 (75.75–95.00)	83.00 (73.00–94.00)	84.00 (76.00–94.00)	0.663
NIHSS score on admission	5.00 (3.00–9.00)	5.00 (3.00–8.00)	4.00 (3.00–8.00)	6.00 (4.00–11.25)	<0.001
NIHSS score 24 h after thrombolysis	5.00 (2.00–9.00)	4.00 (2.00–8.00)	4.00 (2.00–7.00)	6.50 (3.00–11.00)	<0.001
END, *n*(%)	103 (21.78)	29 (18.59)	22 (13.66)	52 (33.33)	<0.001

CRP/HDL-C ratio, C-reactive protein to high-density lipoprotein cholesterol ratio; WBC, white blood cell count; HGB, hemoglobin; TC, total cholesterol; TG, triglycerides; HDL-C, high-density lipoprotein cholesterol; LDL-C, low-density lipoprotein cholesterol; ALB, albumin; ALT, alanine aminotransferase; AST, aspartate aminotransferase; Cr, creatinine; UA, uric acid; SBP, symptomatic intracranial hemorrhage; DBP, diastolic blood pressure; NIHSS, National Institutes of Health Stroke Scale; END, early neurological deterioration.

### 3.2 Baseline characteristics of patients with and without END

**Figure 2:**
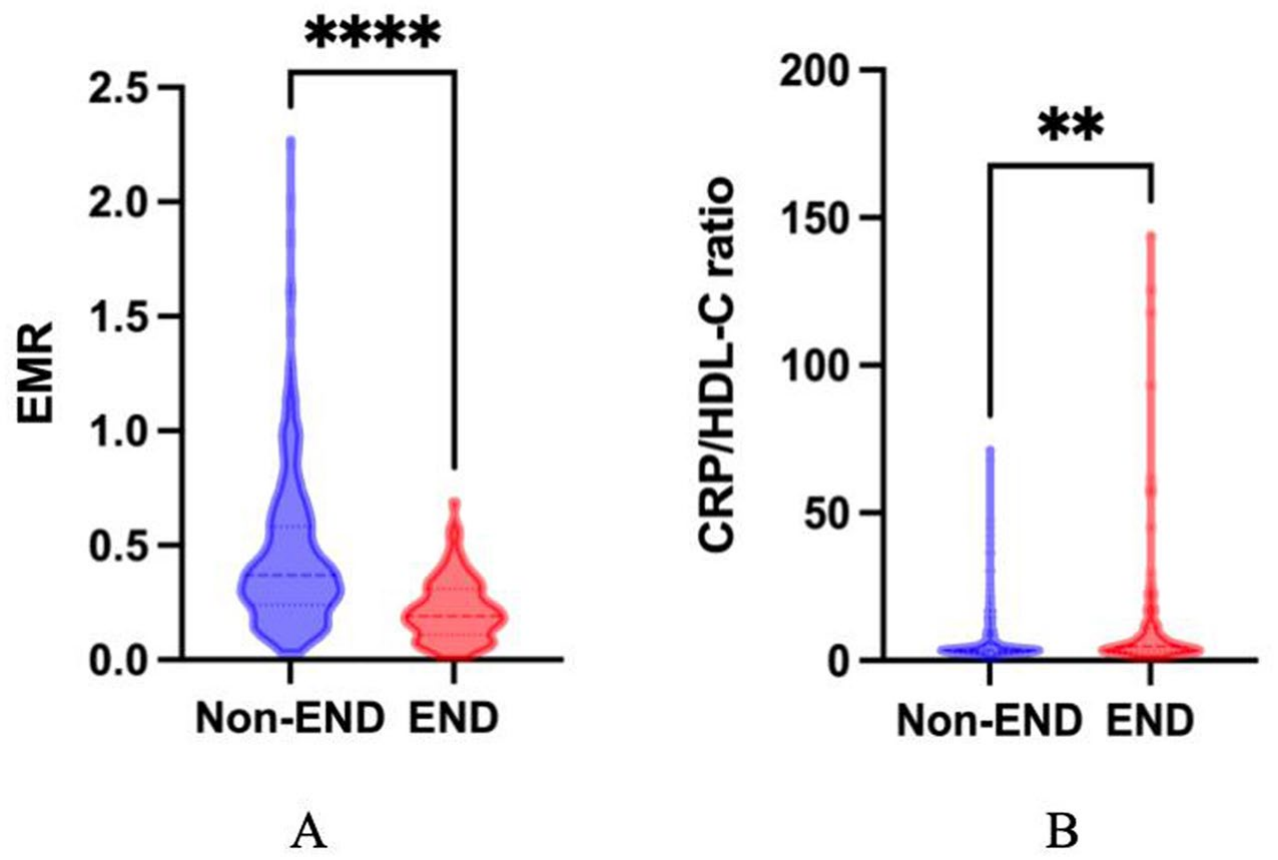
Comparisons of EMR and the CRP/HDL-C ratio between END and non-END patients. The violin plots in the distribution of EMR and CRP/HDL-C ratio between END and non-END patients. **(A)** The violin plot in the distribution of EMR between END and non-END; the mean ± SEM values for the two groups were 0.22 ± 0.01 and 0.45 ± 0.02, respectively. **(B)** The violin plot in the distribution of CRP/HDL-C ratio between END and non-END, the mean ± SEM of the two groups were 13.12 ± 2.44 and 6.00 ± 0.41, respectively. END, early neurological deterioration; EMR, eosinophil-to-monocyte ratio; CRP/HDL-C ratio, reactive protein to high-density lipoprotein cholesterol ratio; **p < 0.01, **** *p* < 0.0001.

Table 3 presents a comparison of characteristics between patients who did or did not experience END. Among those, END was observed in 103 (21.8%) patients. There was a statistically significant difference in EMR (0.37[interquartile range (IQR),0.24–0.58] vs. 0.19[IQR,0.11–0.3]; *p* < 0.001) and CRP/HDL-C ratio (3.62 [IQR, 2.79–5.26] vs. 4.97 [3.07–11.00]; *p* < 0.001) between the two groups. In addition, the systolic blood pressure (SBP) and diastolic blood pressure (DBP) values and white blood cell counts in the END group were higher than those in the normal END group. Additionally, patients with END exhibited higher NIHSS scores on admission than patients without END. Figure 2 shows a comparison of EMR and the CRP/HDL-C ratio between END and non-END patients.

**Table 3:** Comparison of characteristics between patients who did and did not experience END.

Variables	Total (*n* = 473)	Non-END, (*n* = 370)	END, (*n* = 103)	*p* value
Demographic data				
Age (years)	68 (58–75)	68 (59–75)	67 (57–75)	0.419
Sex (male, *n*%)	297 (62.79)	230 (62.16)	67 (65.05)	0.592
Vascular risk factors (*n*%)				
Current smoking	187 (39.53)	152 (41.08)	35 (33.98)	0.192
Current drinking	87 (18.39)	71 (19.19)	16 (15.53)	0.397
Hypertension	305 (64.48)	241 (65.14)	64 (62.14)	0.574
Diabetes	99 (20.93)	76 (20.54)	23 (22.33)	0.693
Coronary heart disease	58 (12.26)	51 (13.78)	7 (6.80)	0.056
Previous stroke	71 (15.01)	52 (14.05)	19 (18.45)	0.270
Atrial fibrillation	40 (8.46)	33 (8.92)	7 (6.80)	0.493
Stroke subtypes, (*n*%)				0.640
Large artery atherosclerosis	125 (26.43)	97 (26.22)	28 (27.18)	
Cardioembolism	45 (9.51)	32 (8.65)	13 (12.62)	
Small artery occlusion	270 (57.08)	215 (58.11)	55 (53.40)	
Other or undetermined	33 (6.98)	26 (7.03)	7 (6.80)	
Laboratory data				
WBC (10^9^/L)	7.09 (5.88–8.62)	6.96 (5.77–8.40)	7.25 (6.27–9.77)	0.005
HGB (g/L)	139.18 ± 17.89	139.33 ± 17.15	138.66 ± 20.40	0.738
Eosinophils (10^9^/L)	0.12 (0.07–0.20)	0.14 (0.08–0.22)	0.09 (0.04–0.14)	<0.001
Monocytes (10^9^/L)	0.39 (0.30–0.50)	0.38 (0.29–0.48)	0.44 (0.34–0.58)	<0.001
EMR	0.32(0.19–0.50)	0.37(0.24–0.58)	0.19(0.11–0.3)	<0.001
TC (mmol/L)	4.41 (3.64–5.11)	4.45 (3.71–5.17)	4.31 (3.59–4.89)	0.103
HDL-C (mmol/L)	1.02 (0.88–1.21)	1.02 (0.89–1.21)	1.00 (0.84–1.18)	0.278
LDL-C (mmol/L)	2.78 (2.21–3.37)	2.79 (2.24–3.41)	2.75 (2.07–3.31)	0.181
TG (mmol/L)	1.39 (0.97–2.09)	1.44 (0.98–2.14)	1.29 (0.93–1.91)	0.106
CRP	3.41 (3.13–6.09)	3.31 (3.13–5.62)	3.81 (3.22–10.66)	<0.001
CRP/HDL	3.73 (2.87–6.07)	3.62 (2.79–5.26)	4.97 (3.07–11.00)	<0.001
ALT (U/L)	19.00 (14.00–26.00)	19.00 (14.00–26.00)	19.50 (15.00–27.00)	0.160
AST (U/L)	22.00 (18.00–27.00)	22.00 (18.00–27.00)	23.50 (18.00–30.00)	0.188
Cr (μmol/L)	73.00 (62.00–88.00)	73.00 (62.00–87.00)	74.00 (63.50–91.00)	0.491
UA (μmol/L)	333.50 (278.00–400.25)	331.00 (278.00–399.00)	338.00 (279.00–401.50)	0.461
Urea (mmol/L)	5.40 (4.56–6.52)	5.35 (4.56–6.52)	5.55 (4.62–6.46)	0.569
SBP (mmHg)	147.00 (134.00–164.00)	146.00 (133.00–161.00)	152.00 (138.00–170.00)	0.044
DBP (mmHg)	84.00 (75.00–94.00)	82.00(75.00–94.00)	88.00(78.50–98.00)	0.008
NIHSS score on admission	5.00 (3.00–9.00)	5.00 (3.00–8.00)	6.00 (4.00–11.00)	<0.001
NIHSS score 24 h after starting thrombolysis	5.00 (2.00–9.00)	3.00 (2.00–6.00)	10.00 (7.50–15.50)	<0.001

END, early neurological deterioration; EMR, eosinophil-to-monocyte ratio; CRP/HDL-C ratio, C-reactive protein to high-density lipoprotein cholesterol ratio; WBC, white blood cell count; HGB, hemoglobin; TC, total cholesterol; TG, triglycerides; HDL-C, high-density lipoprotein cholesterol; LDL-C, low-density lipoprotein cholesterol; ALB, albumin; ALT, alanine aminotransferase; AST, aspartate aminotransferase; Cr, creatinine; UA, uric acid; SBP, symptomatic intracranial hemorrhage; DBP, diastolic blood pressure; NIHSS, National Institutes of Health Stroke Scale.

### 3.3 Logistic regression analysis of END following intravenous thrombolysis therapy

Table 4 illustrates the results of the multiple logistic regression model for END. After matching by age and sex, the EMR levels in the END patients remained lower than those without END (adjusted odds ratio [OR] 0.04 [95% confidence interval (CI), 0.02–0.11]; model 2), while the CRP/HDL-C ratio in the END group was not significantly higher than that in the non-END group (adjusted OR 0.64 [95%CI, 1.33–1.25]; model 2). The same results were obtained for EMR following adjustment for all potential covariates (adjusted OR of 0.03 [95%CI, 0.01–0.14]; model 3). However, after adjusting for all potential covariates, the CRP/HDL-C ratio was significantly higher in patients with END than in those without END (adjusted OR of 3.22 [95%CI, 1.25–8.25]; model 3).

**Table 4:** Multivariate logistic regression to assess the potential relationships of EMR and the CRP/HDL-C ratio with END.

Variables	Model 1	*p* value	Model 2	*p* value	Model 3	*p* value
	OR (95%CI)		OR (95%CI)		OR (95%CI)	
EMR (tertiles)						
T1	1.00 (Reference)		1.00 (Reference)		1.00 (Reference)	
T2	0.34 (0.21 ~ 0.57)	<0.001	0.24 (0.13 ~ 0.43)	<0.001	0.32 (0.13 ~ 0.81)	<0.0016
T3	0.08 (0.04 ~ 0.17)	<0.001	0.04 (0.02 ~ 0.11)	<0.001	0.03 (0.01 ~ 0.14)	<0.001
CRP/HDL-C (tertiles)						
S1	1.00 (Reference)		1.00 (Reference)		1.00 (Reference)	
S2	0.69 (0.38 ~ 1.27)	0.234	0.64 (0.33 ~ 1.25)	0.190	1.28 (0.47 ~ 3.50)	0.637
S3	2.19 (1.30 ~ 3.69)	0.003	2.43 (1.35 ~ 4.40)	0.003	3.22 (1.25 ~ 8.25)	0.015

Model 1: did not adjust other covariates. Model 2: adjusted for age and sex. Model 3: adjusted for age, sex, SBP, DBP, WBC, NIHSS score on admission, NIHSS score 24 h after thrombolysis. EMR tertiles: T1 < 0.24, 0.24 ≤ T2 ≤ 0.42, T3 > 0.42. CRP/HDL-C ratio: S1 < 3.17, 3.17 ≤ S2 ≤ 4.77, S3 > 4.77.

### 3.4 Receiver operating characteristic (ROC) curve analysis for post-thrombolysis END in AIS patients

**Figure 3:**
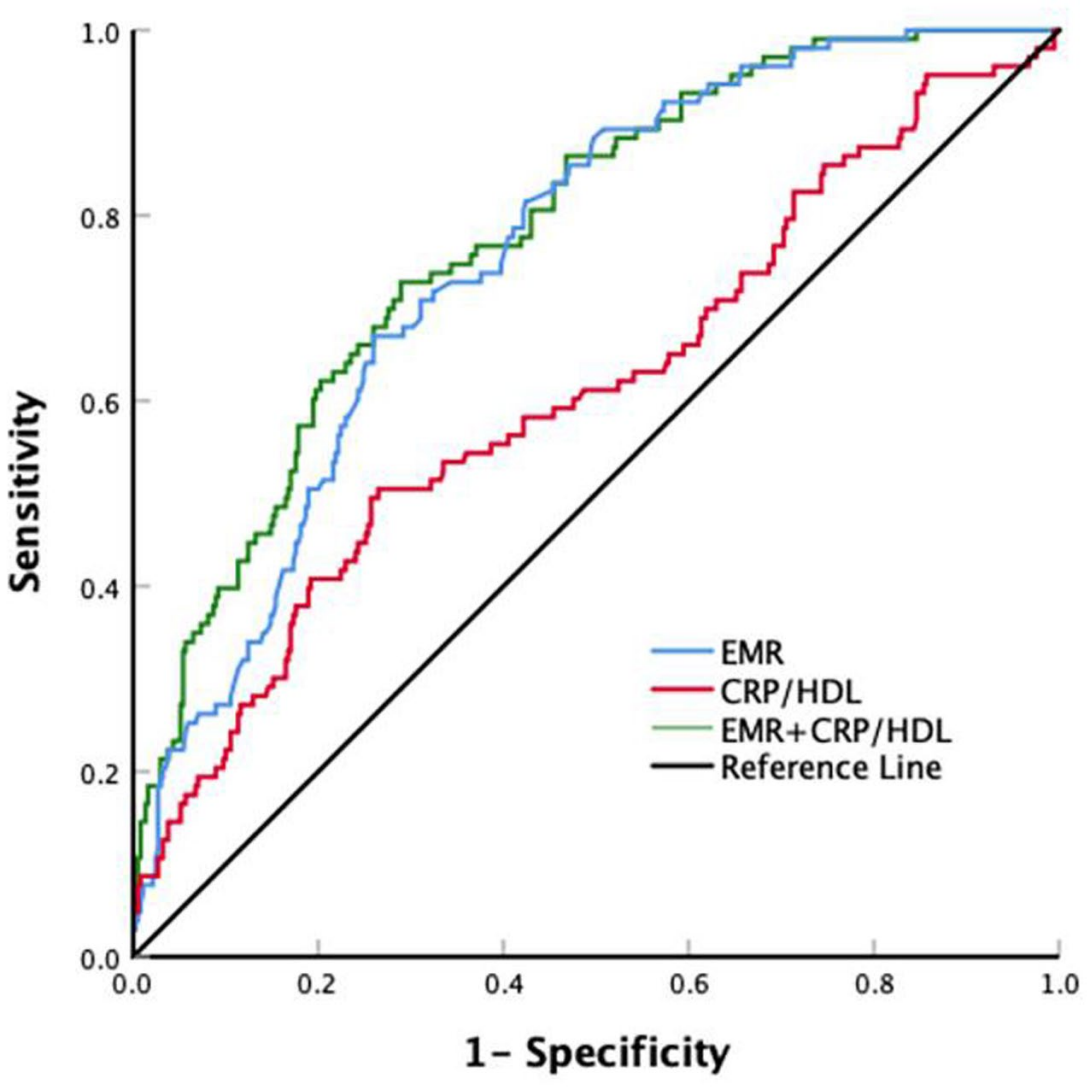
ROC for EMR and the CRP/HDL-C ratio to predict post-thrombolysis END. ROC for EMR, CRP/HDL-C ratio, and EMR + CRP/HDL-C ratio to predict post-thrombolysis END. The blue line represents an AUC of 0.757 for EMR, the red line represents an AUC of 0.610 for the CRP/HDL-C ratio, and the green line represents an AUC of 0.780 for EMR combined with the CRP/HDL-C ratio.

ROC curves analysis was performed to assess the discriminative ability of both EMR and CRP/HDL-C ratio concerning post-thrombolysis END (Figure 3). We observed that the area under the curve (AUC) of EMR was 0.757 (95%CI, 0.709–0.805) and the cutoff value was 0.25, with a sensitivity of 67% and a specificity of 74.1%. For the CRP/HDL-C ratio, the AUC was 0.610 (95%CI,0.545–0.675), with a cutoff value of 5.02, sensitivity of 49.5%, and specificity of 74.3%. Overall, to predict post-thrombolysis END, the AUC of EMR was superior to that of CRP/HDL-C. However, the combined AUC of EMR and the CRP/HDL-C ratio was 0.780 (95% CI, 0.732–0.828), with a cutoff value of 0.16, sensitivity of 72.8%, and specificity of 71.1%, ultimately rendering both together a more accurate predictor of post-thrombolysis END.

## 4 Discussion

In this study, we found that the prevalence of post-thrombolysis END was 21.8%, which is similar to rates reported by previous research (25, 26). Furthermore, we demonstrated that a lower EMR level and a higher CRP/HDL-C ratio are independently associated with post-thrombolysis END in AIS patients undergoing intravenous thrombolysis. This study revealed that EMR and the CRP/HDL-C ratio may be used to predict the post-thrombolysis END.

In recent years, neuroinflammation has received increasing attention, and many studies have confirmed that inflammatory mechanisms play a crucial role in the pathogenesis and progression of AIS (27, 28). Peripheral leukocytes are indispensable inflammatory cells, among which eosinophils are considered to induce the activation of the M2 phenotype microglia by secreting cytokines, growth factors, and chemokines, such as interleukin (IL)-4 and IL-13, thereby exerting neuroprotective effects in AIS (29). Meanwhile inflammatory mediators released by monocytes, such as chemokines, intercellular adhesion molecule 1, IL-1, IL-6, IL-8, and tumor necrosis factor, promote inflammation, leading to AIS (30). A previous study found that AIS triggers a decrease in eosinophil counts and an increase in monocyte counts (14). A strong stress response and eosinophilic infiltration may be important mechanisms of eosinophilpenia after stroke (31, 32). Furthermore, Zhao et al. demonstrated that lower eosinophil counts were associated with poor prognosis in patients with AIS (33). EMR is a novel biomarker that reflects the comprehensive application value of eosinophils and monocytes; however, the association between EMR and END after intravenous thrombolysis in patients with AIS has not been clarified. Chen et al. found that the reduction in EMR levels was caused by a decrease in eosinophil counts, while the difference in monocyte counts between the good prognosis group and the poor prognosis group was not statistically significant (34). However, in this study, we found that the differences in eosinophils and monocytes between the END group and non-END group patients were statistically significant, indicating that the low level of EMR is due to the decrease in eosinophil count and the an increase in monocyte count. As expected, a lower EMR was independently positively associated with END in AIS patients undergoing intravenous thrombolysis. The finding complements the role of EMR in cerebrovascular diseases and provides new ideas for clinical practice.

As is well known, intracranial atherosclerosis is a crucial etiological component of AIS, and inflammation and dyslipid metabolism are considered the basis of pathophysiology and for the progression of atherosclerosis. CRP is a non-specific inflammation biomarker, elevated in patients with AIS and regulated by pro-inflammatory cytokines (35). It has been established that elevated levels of CRP are associated with increased in-hospital mortality rates and long-term poor functional outcomes in patients with AIS (36). Recently, a meta-analysis revealed that high CRP levels are associated with a poor prognosis at 3 months in patients with AIS who were treated with intravenous thrombolysis (37). As a beneficial cholesterol in the human body, HDL-C may reduce thrombosis by hindering platelet activation and aggregation. Meanwhile, it also promotes the repair of damaged vascular endothelium (20). A large prospective study found that HDL-C levels in AIS patients with diabetes were inversely correlated with the risk of recurrent AIS (38). Yeh et al. demonstrated that low levels of HDL-C (≤ 35 mg/dL) are associated with greater stroke severity and poor clinical prognosis in AIS patients (39). The CRP/HDL-C ratio can reflect the interaction between inflammatory states and lipid metabolism, and may more accurately predict the prognosis of AIS independently than a single inflammatory marker or a single lipid marker. A recent study showed that an elevated high-sensitivity CRP/HDL-C ratio is associated with the development and severity of coronary heart disease (OR 1.31[95% CI, 1.05–1.64]) (40). A large cohort study revealed that the high-sensitivity CRP/HDL-C ratio is positively correlated with stroke (OR: 1.17 [95% CI, 1.02, 1.35]), and the higher this ratio, the greater the likelihood of stroke (41). Nevertheless, there is limited research on the role of the CRP/HDL-C in ischemic stroke outcomes. The present study demonstrated that the CRP/HDL-C ratio may be independently associated with post-thrombolysis END. As such, early intervention, according to the CRP/HDL-C ratio, may reduce the incidence of post-thrombolysis END.

In this study, we also found that patients with END have significantly higher SBP and DB*p* values and NIHSS scores on admission. A previous study reported that the risk of AIS in hypertensive patients was up to 78.9%; meanwhile, hypertension is also thought to be associated with a poor prognosis in AIS (42). Wei et al. found that a SBP of >185 mmHg or DBP of >110 mmHg in the first 24 h after starting intravenous thrombolytic therapy for acute subcortical infarction is independently associated with unexplained END (43). Recent research has also concluded that electrolyte imbalance (specifically, elevated Na + and Ca^2+^ concentrations) is associated with END in AIS patients with hypertension (44). Therefore, aggressive blood pressure control before stroke and appropriate blood pressure control after intravenous thrombolysis may reduce the incidence of END. It is well known that the NIHSS score is used to assess the severity of AIS. In this study, patients with a high NIHSS score on admission were more likely to develop END, which may be attributed to a greater incidence of cerebral edema and hemorrhagic transformation in such patients (45).

The main findings of this study were as follows: (1) there were statistically significant differences in the EMR and the CRP/HDL-C ratio between the END and non-END groups; (2) the multiple logistic regression model showed that the EMR and the CRP/HDL-C ratio are independently associated with END in patients with AIS; and (3) ROC curve analysis revealed that EMR exhibits greater accuracy in predicting post-thrombolysis END than the level of CRP/HDL-C ratio. However, inevitably, this study also has several limitations. First, the sample size of this study was relatively small. Second, because all the patients participating in this study were from the same hospital, the results we obtain have certain limitations. Furthermore, there was no dynamic examination of EMR to the CRP/HDL-C ratio; according to reports, the composite inflammation ratio may change significantly during hospitalization. Our future research needs to dynamically monitor the composite inflammation ratio. Finally, we did not explore the role of interactions between eosinophils and monocytes, along with those between CRP and HDL-C, in the pathogenesis of AIS. More basic experiments are needed in the future to validate the role of these indicators in AIS.

## 5 Conclusion

In conclusion, this study demonstrates that a lower EMR level and a higher CRP/HDL-C ratio are independent predictors of early neurological deterioration (END) after thrombolysis in acute ischemic stroke. Notably, the combined assessment of both biomarkers showed additive predictive value, suggesting they may reflect distinct yet complementary pathways involving systemic inflammation, stress response, and metabolic dysregulation. Therefore, early and serial monitoring of EMR and CRP/HDL-C ratio could enhance risk stratification and help identify patients who may benefit from closer monitoring or adjunctive therapies targeting inflammation and metabolic status. Further prospective and multicenter studies are warranted to validate their clinical utility.

## Data Availability

The raw data supporting the conclusions of this article will be made available by the authors, without undue reservation.
